# Assessment of Disability and Depression Following Amputation Among Adults in Korea

**DOI:** 10.1001/jamanetworkopen.2023.20873

**Published:** 2023-06-29

**Authors:** Wonyoung Jung, Miso Kim, Hong Jin Jeon, Won Hyuk Chang, Jung Eun Yoo, Kyungdo Han, Dong Wook Shin

**Affiliations:** 1 Department of Family Medicine, Kangdong Sacred Heart Hospital, Hallym University, Seoul, Korea; 2 Department of Medicine, Sungkyunkwan University School of Medicine, Seoul, Korea; 3 Department of Family Medicine/Supportive Care Center, Samsung Medical Center, Sungkyunkwan University School of Medicine, Seoul, Korea; 4 Depression Center, Department of Psychiatry, Samsung Medical Center, Sungkyunkwan University School of Medicine, Seoul, Korea; 5 Department of Clinical Research Design & Evaluation, Samsung Advanced Institute for Health Science & Technology, Sungkyunkwan University, Seoul, Korea; 6 Department of Physical & Rehabilitation Medicine, Samsung Medical Center, Sungkyunkwan University School of Medicine, Seoul, Korea; 7 Department of Family Medicine, Seoul National University Hospital Healthcare System Gangnam Center, Seoul, Korea; 8 Department of Statistics and Actuarial Science, Soongsil University, Seoul, Korea

## Abstract

This nationwide, population-based, retrospective cohort study assesses the risk of depression following amputation among adults in Korea.

## Introduction

Amputation is a life-changing experience that affects physical ability, body image, quality of life, and stress resilience, all of which are factors associated with depression.^1,2^ However, most previous studies^3,4,5^ examining the risk of depression among people with amputation have been conducted in special populations (eg, veterans), confined to traumatic amputation, and limited by cross-sectional designs. Hence, we investigated the risk of subsequent depression after all-cause amputation in this nationwide, population-based, retrospective cohort study in Korea. We hypothesized that amputation might be associated with increased risk of depression, and that this risk could be higher for individuals experiencing disability resulting from amputation.

## Methods

This study was approved by the institutional review board of the Samsung Medical Center and adhered to the Strengthening the Reporting of Observational Studies in Epidemiology (STROBE) reporting guideline. This study enrolled individuals with amputations (aged ≥20 years) and a comparison group matched in a 1:3 ratio for age, sex, and year of amputation using data from the Korean National Health Insurance Service database from 2010 to 2018 (eFigure in Supplement 1). The group with amputations was further categorized on the basis of their disability status and severity (eTable in Supplement 1). The primary outcome was incident depression based on codes from the *International Statistical Classification of Diseases and Related Health Problems, Tenth Revision (ICD-10)*. Cox hazard regression models were used to examine hazard ratios (HRs) of depression after adjusting for potential confounders. Additional methods can be found in the eMethods in Supplement 1.

## Results

The cohort consisted of 21 633 individuals with amputations (mean [SD] age, 53.4 [12.1] years; 17 134 men [79.2%]) and 71 895 individuals without amputations (mean [SD] age, 52.8 [11.8] years; 58 054 men [80.8%]). Among individuals with amputations, 40 (0.2%) had a nontraumatic amputation (*ICD-10* code Z89; acquired absence of limb), and 21 593 (99.8%) had a traumatic amputation with 1 of the following *ICD-10* codes: S48 (shoulder joint; 50 patients [0.23%]); S58 (forearm; 95 patients [0.44%]); S68 (wrist or finger; 20 811 patients [96.20%]); S78 (hip; 30 patients [0.14%]); S88 (knee; 131 patients [0.61%]); or S98 (ankle or toe; 476 patients [2.20%]). Compared with the comparison group, the group with amputations had higher baseline prevalence of smoking, alcohol consumption, rural residency, lower income, and diabetes. This trend was more pronounced among individuals with amputations with a registered disability who also had a higher prevalence of hypertension and dyslipidemia.

During a mean (SD) follow-up of 4.9 (2.6) years for people in the amputation group and 5.1 (2.6) years for people in the comparison group, individuals in the amputation group had a 20% increased risk of depression (adjusted HR [aHR], 1.20; 95% CI, 1.15-1.26) after adjusting for potential confounders (Table). Further analysis that classified amputation by disability demonstrated a 17% increased risk of depression in individuals with amputation without a registered disability (aHR, 1.17; 95% CI, 1.12-1.22) and 57% increased risk of depression for individuals with amputation with registered disabilities (aHR, 1.57; 95% CI, 1.40-1.75) compared with the comparison group. In addition, individuals with amputations with mild registered disabilities exhibited a 46% increased risk of depression (aHR, 1.46; 95% CI, 1.29-1.65) and individuals with amputations with severe registered disabilities exhibited a 113% increase in risk of depression (aHR, 2.13; 95% CI, 1.69-2.69). A steep increase in depression incidence (up to 20%) within 2 years after amputation was found in people with severe disabilities (Figure).

**Table.** Risk of Subsequent Depression by Amputation and Disability Status^a^

Group	Age, mean (SD), y	Sex, patients, No. (%)		Current smoker, patients, No. (%)	Heavy alcohol consumption, patients, No. (%)^b^	Diagnosis of depression, No.	IR 1000 person-years	Model 1, crude HR (95% CI)	Model 2, aHR (95% CI)^c^	Model 3, aHR (95% CI)^d^
		Male	Female							
Matched comparison group (n = 71 895)	52.8 (11.8)	58 054 (80.8)	13 841 (19.2)	22 812 (31.7)	7841 (10.9)	7447	20.24	1 [Reference]	1 [Reference]	1 [Reference]
Amputation group (n = 21 633)	53.4 (12.1)	17 134 (79.2)	4499 (20.8)	7665 (35.4)	2941 (13.6)	2812	26.44	1.31 (1.25-1.37)	1.23 (1.18-1.29)	1.20 (1.15-1.26)
Amputation without registered disability (n = 19 990)	52.9 (12.1)	15 736 (78.7)	4254 (21.3)	7096 (35.5)	2704 (13.5)	2471	25.13	1.24 (1.19-1.30)	1.19 (1.14-1.25)	1.17 (1.12-1.22)
Amputation with any registered disability (n = 1643)	59.0 (10.8)	1398 (85.1)	245 (14.9)	569 (34.6)	237 (14.4)	341	42.48	2.10 (1.88-2.34)	1.61 (1.45-1.80)	1.57 (1.40-1.75)
Amputees with mild registered disability (n = 1360)	59.3 (10.5)	1156 (85)	204 (15)	473 (34.8)	196 (14.4)	269	39.90	1.97 (1.74-2.22)	1.50 (1.33-1.70)	1.46 (1.29-1.65)
Amputees with severe registered disability (n = 283)	57.5 (12.1)	242 (85.5)	41 (14.5)	96 (33.9)	41 (14.5)	72	56.03	2.77 (2.20-3.50)	2.22 (1.76-2.80)	2.13 (1.69-2.69)

Abbreviations: aHR, adjusted hazard ratio; HR, hazard ratio; IR, incidence rate.

^a^ For details on registered disabilities in Korea, refer to the eTable in Supplement 1.

^b^ Heavy alcohol consumption was defined as 30 g/day or more.

^c^ Model 2 was adjusted for age, sex, and Charlson Comorbidity Index.

^d^ Model 3 was adjusted for age, sex, Charlson Comorbidity Index, income, area of residence, diabetes, hypertension, dyslipidemia, smoking, alcohol consumption, and physical activity.

**Figure.**
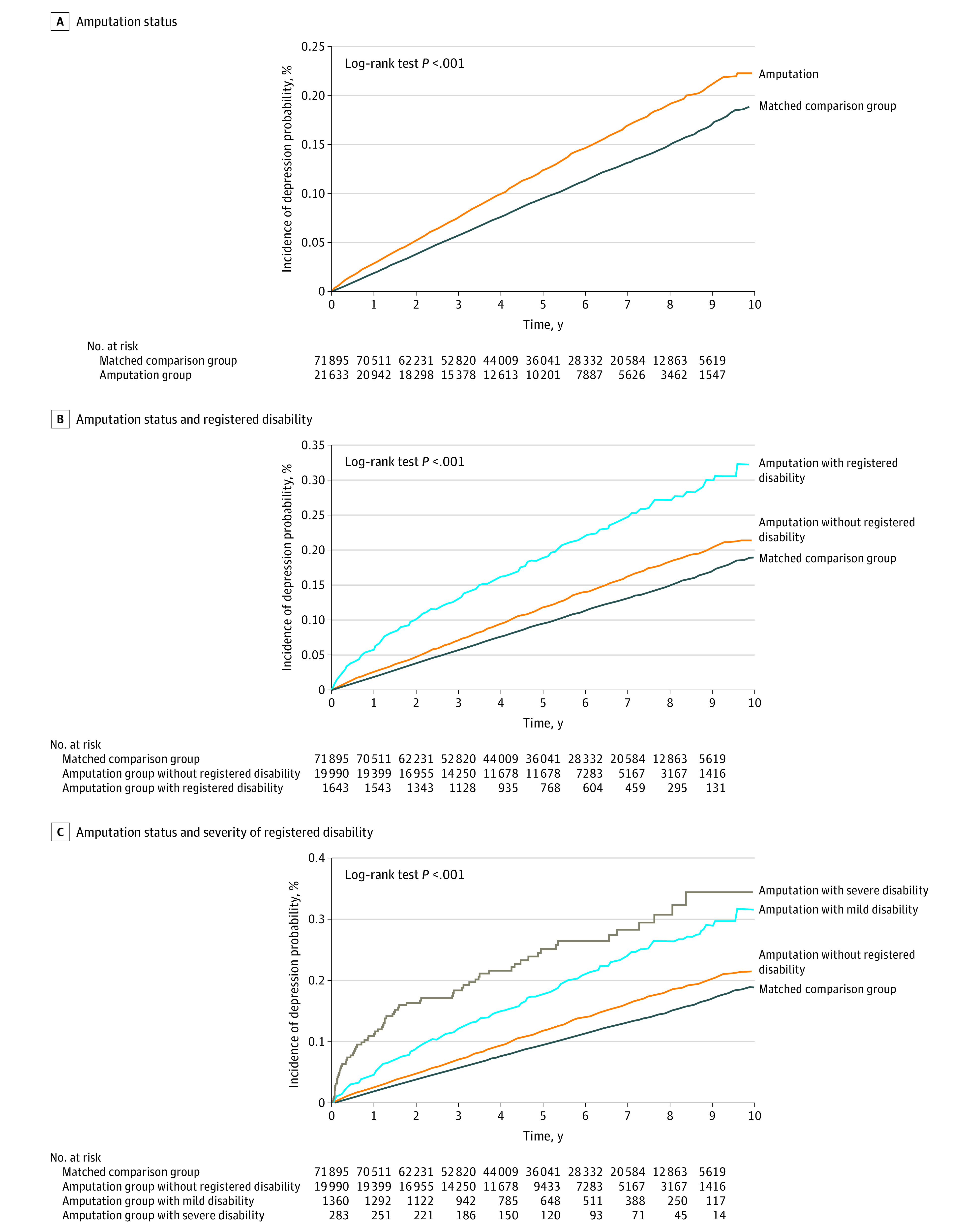
Estimated Incidence Probability of Depression Subsequent to Amputation Figure shows Kaplan-Meier curves displaying the estimated incidence probability of subsequent depression by amputation status (A), by amputation status and registered disability (B), and by amputation status and severity of registered disability (C).

## Discussion

In this cohort study in Korea, we found an increased risk of depression after amputation, emphasizing the importance of disability status and severity in evaluating depression risk among people with amputation. Amputation generates an irreversible physical condition that may lead to feelings of loss of control, which can affect the risk of depression. In addition, depression could lead to further functional disability and poor compliance with rehabilitation.^6^ Therefore, proactive rehabilitation including psychosocial support for adjustment to amputation is needed. The limitations of our study include its observational design and possible underdetection of depression. To mitigate the potential psychological effects of amputation, it is advisable to conduct screenings and provide psychological support for individuals who are at risk of severe disability resulting from the amputation, particularly during the early stages following the amputation.

## Notes

Supplement 1. **eMethods. **Supplemental Methods **eFigure.** Flowchart of Study Population **eTable.** Definitions of Registered Disability Severity From Amputation
eReferences

Supplement 2.
Data Sharing Statement
