# Supplementary material for: Assessment of Disability and Depression Following Amputation Among Adults in Korea
Source: JAMA Netw Open. 2023 Jun 29;6(6):e2320873. doi: 10.1001/jamanetworkopen.2023.20873 (PMC10311386; doi:10.1001/jamanetworkopen.2023.20873)
Supplement: Supplement 1. — eMethods. Supplemental Methods eFigure. Flowchart of Study Population eTable. Definitions of Registered Disability Severity From Amputation eReferences [file jamanetwopen-e2320873-s001.pdf]

Supplemental Online Content

Jung W, Kim M, Jeon HJ, et al. Assessment of disability and depression following amputation among adults in Korea. *JAMA Netw Open*. 2023;6(6):e2320873. doi:10.1001/jamanetworkopen.2023.20873

eMethods. Supplemental Methods

eFigure. Flowchart of Study Population

eTable. Definitions of Registered Disability Severity From Amputation

eReferences

This supplemental material has been provided by the authors to give readers additional information about their work.

© 2023 Jung W et al. *JAMA Network Open.*

eMethods. Supplemental Methods

Data source and study setting

This study is based on the database provided by the National Health Insurance Service (NHIS) in Korea. The NHIS provides medical insurance to about 97% of the Korean population, additional to managing administration of medical aid for the population of patients with the lowest income levels. Korea's NHIS provides general health screening for individuals who are employees and for all people aged 40 and above every two years, which is conducted by medical institutions throughout Korea.[1] The screening program contains anthropometric measurements, questionnaires about medical and social history, and laboratory tests. In addition, the medical treatment database (based on medical bills claimed by medical service providers for medical expenses) can be linked with the health examination database. Accordingly, the NHIS retains an extensive health information dataset on the entire Korean population.

Study population

We identified 59,392 subjects who underwent amputation from January 1, 2010, to December 31, 2018, and matched them by age, sex, and index year of amputation to select a comparison group in a 1:3 ratio. The definition of amputation was based on the following international classification of disease 10th Edition (ICD-10) codes: ICD-10 codes: Z89 (acquired absence of limb), S48 (traumatic amputation of shoulder joint), S58 (traumatic amputation of forearm), S68 (traumatic amputation of wrist, finger), S78 (traumatic amputation of hip), S88 (traumatic amputation of knee), and S98 (traumatic amputation of ankle, toe).

© 2023 Jung W et al. *JAMA Network Open.*

Among the amputation group and the comparison group defined by the above, we included only subjects who underwent a general health examination preceding the index date within two years, thereby enabling the incorporation of diverse covariables in our analysis. Next, among the amputation group (n = 27,832) and the comparison group (n = 88,900), we excluded subjects who were under 20 years of age (n = 22 and 14, respectively), subjects with previous diagnoses of depression (n = 5,176 and 13,749, respectively), and those with missing data (n = 1,001 and 3,242, respectively). Finally, a total of 21,633 and 71,895 participants were included in the amputation group and the comparison group, respectively. (**see flowchart of study population for detail**)

Definition of disability

People with amputation are eligible for Korea's national disability registration (KNDR) system from the index date of amputation. To be registered as having extremities disability, documentation of the amputation diagnosis based on physical examinations and X-rays by specialist physicians is required.[2] Disability severity is classified into 6 grades by predefined criteria during periods of study. In general, amputation of proximal sites equates to severity in grades of disability. For example, grade 1 (most severe) disability due to upper extremity amputation is defined by amputation above the wrist joint of both arms. Grade 6 (least severe) disability due to upper extremity amputation, however, is defined by amputation of one thumb above the interphalangeal joint. Because KNDR brings access to social welfare benefits such as disability pension based on the determined disability severity,[3] almost all people with amputation register in the KNDR system and disability severity information can be regarded as accurate. **(see**

© 2023 Jung W et al. *JAMA Network Open.*

definitions of registered disability severity from amputation for detail)

Study outcomes and follow-up

The primary outcome is newly diagnosed depression based on ICD-10 codes including F32 (depressive episode) and F33 (recurrent depressive disorder), as defined in previous epidemiological studies conducted in Korea.[4] Participants were followed from the index date of amputation to the date of newly diagnosed depression or a censor date (e.g., outmigration to another countries) or until the end of the study period (December 31, 2019), whichever came first.

Covariates

Sociodemographic information including age, sex, residential area, and income levels of participants were provided by the NHIS database. Information on lifestyle behaviors (smoking, alcohol consumption, and physical activity), anthropometric measurements, and laboratory test results (total cholesterol, triglycerides, fasting glucose, and estimated glomerular filtration rate [eGFR]) was derived from the results of general health screening examinations preceding the index date of amputation within two years.

Comorbidities of participants were identified based on claims and prescription information prior to the index date as follows: hypertension (ICD-10 codes [I10.x-I13.x and I15.x], or being on antihypertensive medication or having blood pressure ≥ 140/90 mmHg), diabetes mellitus (DM) (ICD-10 codes [E11.x-E14.x] with antidiabetic medications, or a fasting glucose level ≥ 126 mg/dL), and dyslipidemia (ICD-10 code E78.x with lipid-lowering medication, or total

© 2023 Jung W et al. *JAMA Network Open.*

cholesterol level ≥ 240 mg/dL). Participants' Charlson Comorbidity Index (CCI) was also calculated based on ICD-10 codes.[5]

Statistical analysis

Mean ± standard deviation (SD) for continuous variables and number (percentage) for categorical variables were used for descriptive analysis. Cox proportional hazards regression analysis was performed to calculate hazard ratios (HRs) and 95% confidence intervals (CIs) for depression risk among people with amputation relative to the matched comparison group. We obtained HRs in three steps: model 1 unadjusted, model 2 adjusted for age, sex, and CCI, and model 3 additionally adjusted for place of residence, income level, presence of diabetes mellitus, hypertension, dyslipidemia, smoking, alcohol consumption, and level of physical activity. In addition, people with amputation were classified into (1) amputation without disability, (2) amputation with mild disability (grades 4-6), and (3) amputation with severe disability (grades 1-3). Finally, to evaluate potential effects modification by age and sex, P for interaction was calculated using stratified analysis.

Statistical analyses were performed using SAS version 9.4 (SAS Institute Inc., Cary, NC, USA), and a P-value < 0.05 was considered statistically significant.

© 2023 Jung W et al. *JAMA Network Open.*

eFigure. **Flowchart of Study Population**

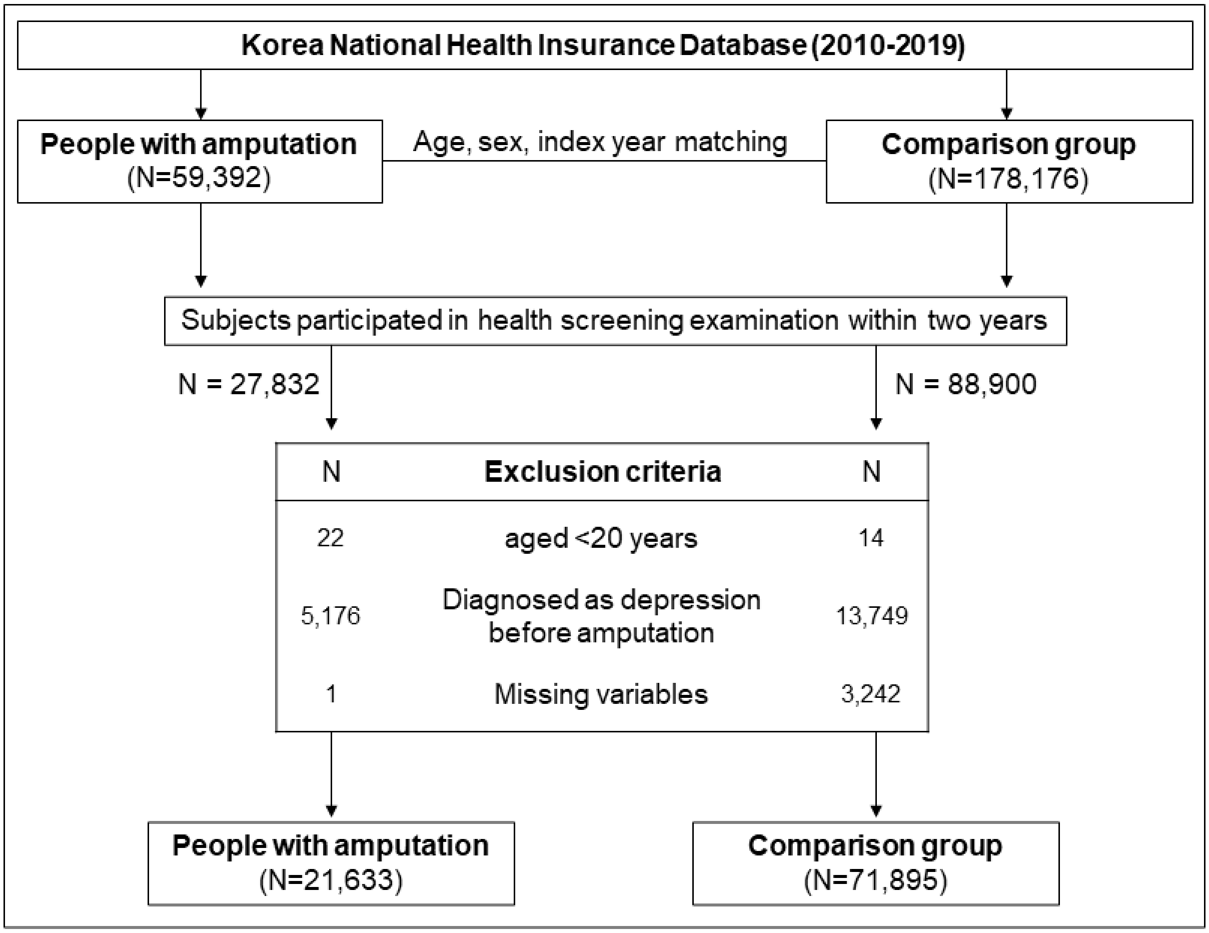

© 2023 Jung W et al. *JAMA Network Open.*

eTable. **Definitions of Registered Disability Severity From Amputation**

Definitions of registered disability severity from upper extremity amputation

Grade		Definition
Level	Number	
1	1	Amputation above the wrist joint of both arms
2	1	Amputation of both thumbs above IP joint and all 2nd to 5th fingers above PIP joint
	2	Amputation above the elbow joint of one arm
3	1	Amputation of both thumbs above IP joint and 2nd finger above PIP joint
	2	Amputation of one thumb above IP joint and all 2nd to 5th fingers above PIP joint
4	1	Amputation of both thumbs above IP joint
	2	Amputation of one thumb above IP joint and 2nd finger above PIP joint
	3	Amputation of one thumb above IP joint and other two fingers above PIP joint
5	1	Amputation of one thumb above IP joint and any other one finger above PIP joint
	2	Amputation of one thumb above MCP joint
	3	Amputation of three fingers above PIP joint including 2nd finger
6	1	Amputation of one thumb above IP joint
	2	Amputation of two fingers above PIP joint including 2nd finger
	3	Amputation of all 3rd to 5th fingers of one hand above PIP joint

IP, Interphalangeal; PIP, proximal interphalangeal; MCP, metacarpophalangeal

© 2023 Jung W et al. *JAMA Network Open.*

Definitions of registered disability severity from lower extremity amputation

Grade		Definition
Level	Number	
1	2	Amputation of both legs above the knee joint
2	3	Amputation of both legs above the ankle joint
3	3	Amputation of both legs above the transverse tarsal joint (Chopart joint)
	4	Amputation of one leg above the knee joint
4	4	Amputation of both legs above the tarsometatarsal joint (Lisfranc joint)
	5	Amputation of one leg above the ankle joint
5	4	Amputation of both big toes above the interphalangeal joint and 2nd to 5th toes above the proximal interphalangeal joint
	5	Amputation of one leg above the transverse tarsal joint (Chopart joint)
6	4	Amputation of one leg above the tarsometatarsal joint (Lisfranc joint)

© 2023 Jung W et al. *JAMA Network Open.*

eReferences

1. Shin DW, Cho J, Park JH, Cho B. National General Health Screening Program in Korea: history, current status, and future direction. *Precis Future Med*. 3 2022;6(1):9-31. doi:10.23838/pfm.2021.00135
2. Shin DW, Lee JW, Jung JH, et al. Disparities in Cervical Cancer Screening Among Women With Disabilities: A National Database Study in South Korea. *J Clin Oncol*. Sep 20 2018;36(27):2778-2786. doi:10.1200/jco.2018.77.7912
3. Number of Registered Persons with Disabilities and Disability Pension Recipients, Ministry of Health and Welfare, Republic of Korea. 2022;(April 5, 2022)
4. Park MJ, Yoo J, Han K, et al. High body weight variability is associated with increased risk of depression: a nationwide cohort study in South Korea. *Psychological medicine*. Mar 8 2022:1-9. doi:10.1017/s003329172200040x
5. Kim KH. [Comparative study on three algorithms of the ICD-10 Charlson comorbidity index with myocardial infarction patients]. *J Prev Med Public Health*. Jan 2010;43(1):42-9. doi:10.3961/jpmph.2010.43.1.42

© 2023 Jung W et al. *JAMA Network Open.*
