# Supplementary material for: Assessment of Disability and Depression Following Amputation Among Adults in Korea
Source: JAMA Netw Open. 2023 Jun 29;6(6):e2320873. doi: 10.1001/jamanetworkopen.2023.20873 (PMC10311386; doi:10.1001/jamanetworkopen.2023.20873)
Supplement: Supplement 2. — Data Sharing Statement [file jamanetwopen-e2320873-s002.pdf]

Data Sharing Statement

Jung. Assessment of Disability and Depression Following Amputation Among Adults in Korea. *JAMA Netw Open*. Published June 29, 2023. doi:10.1001/jamanetworkopen.2023.20873

Data
Data available: Yes
Data types: Deidentified participant data
How to access data: https://nhiss.nhis.or.kr/
When available: With publication

Supporting Documents
Document types: None

Additional Information
Who can access the data: Researchers whose proposed use of the data has been approved
Types of analyses: For a specified purpose
Mechanisms of data availability: The data will be made available upon request and approval of a proposal by the National Health Insurance System Database
